# Elevated 4β‐hydroxycholesterol/cholesterol ratio in anorexia nervosa patients

**DOI:** 10.1002/prp2.430

**Published:** 2018-09-11

**Authors:** Kristine Hole, Petra L. Heiberg, Caroline Gjestad, Lise L. Mehus, Øyvind Rø, Espen Molden

**Affiliations:** ^1^ Center for Psychopharmacology Diakonhjemmet Hospital Oslo Norway; ^2^ Department of Medicinal Biochemistry Diakonhjemmet Hospital Oslo Norway; ^3^ Regional Department for Eating Disorders Division of Mental Health and Addiction Oslo University Hospital Oslo Norway; ^4^ Division of Mental Health and Addiction Institute of Clinical Medicine University of Oslo Oslo Norway; ^5^ Department of Pharmaceutical Biosciences School of Pharmacy University of Oslo Oslo Norway

**Keywords:** 4β‐hydroxycholesterol, anorexia nervosa, BMI, CYP3A

## Abstract

Recent studies have shown that the cytochrome P450 (CYP) 3A phenotype marker 4β‐hydroxycholesterol/cholesterol (4βOHC/C) ratio is negatively correlated with body weight in healthy volunteers, and that obese patients have lower 4βOHC levels than healthy controls. However, 4βOHC/C ratio in underweight patients has yet to be reported. The aim of this study was to examine potential differences in CYP3A activity between underweight patients with anorexia nervosa and normal‐weight volunteers by measuring plasma 4βOHC/C ratio. Furthermore, we wished to describe any association between body mass index (BMI) and 4βOHC/C ratio in underweight patients. A total of 20 underweight patients and 16 normal‐weight volunteers were included in the study, all females. Underweight patients had a median 4βOHC/C ratio (molar ratio × 10^−5^) of 2.52 (range, 0.90–11.3) compared to 1.29 (0.56–2.09) in normal‐weight subjects (Mann‐Whitney *P *= 0.0005). 4βOHC/C ratio was negatively correlated with BMI in underweight patients (*r *= −0.56, *P *= 0.011), and in the whole study population (*r *= −0.67, *P *< 0.0001). This suggests that the negative correlation between 4βOHC/C and BMI, which has previously been reported between 4βOHC/C and body weight in healthy volunteers, extends to underweight patients. The findings indicate that CYP3A activity increases with decreasing BMI, resulting in higher CYP3A activity in underweight patients compared to normal‐weight subjects. The potential clinical relevance of this needs to be studied further by comparing pharmacokinetics of drugs subjected to CYP3A‐mediated metabolism in underweight vs. normal‐weight individuals.

**Abbreviations**
4βOHC/C: 4β‐hydroxycholesterol/cholesterol
BMI: body mass index
CYP: cytochrome P450

## 1 INTRODUCTION

Cytochrome P450 (CYP) 3A enzymes play a major role in the metabolism of about 30% of clinically used drugs.^1^ Substantial inter‐individual variability in CYP3A activity exists due to a combination of genetic, environmental, and endogenous factors.^2^ CYP3A phenotype can be measured by the endogenous biomarker 4β‐hydroxycholesterol (4βOHC), which is metabolized from cholesterol by CYP3A4 and CYP3A5, the two most important CYP3A enzymes in humans.^3^, ^4^ Variations in cholesterol concentration have been found to explain about 10% of 4βOHC variation,^5^ and 4βOHC/cholesterol (4βOHC/C) ratio is preferable to 4βOHC as CYP3A biomarker in patient groups where cholesterol levels are abnormal.^6^

Recent studies have shown that body weight is negatively correlated with 4βOHC/C ratio in healthy volunteers,^7^ and that obese patients have lower 4βOHC levels than healthy controls.^8^ Studies on the clearance of a number of other CYP3A substrates suggest that CYP3A activity is reduced by 10‐35% in obese patients.^9^ Furthermore, Ulvestad et al. reported strong negative correlation between body mass index (BMI) and CYP3A protein expression in liver and intestines.^10^ Altogether, this indicates that CYP3A activity decreases with increasing body weight. However, 4βOHC/C ratio in underweight patients is yet to be reported, and it is not known whether the correlation between BMI and CYP3A activity extends to underweight patients.

The aim of our study was to examine potential differences in CYP3A activity between underweight patients with anorexia nervosa and normal‐weight volunteers by measuring plasma 4βOHC/C ratio. Furthermore, we wished to describe any association between BMI and 4βOHC/C ratio in underweight patients.

## 2 MATERIALS AND METHODS

### 2.1 Subjects

Patients with severe anorexia nervosa (n* *= 20) were included from an inpatient unit at the Regional Department for Eating Disorders, Division of Mental Health and Addiction, Oslo University Hospital, Norway, from May 2012 to September 2013. Inclusion criteria were (i) anorexia nervosa diagnosis according to DSM‐IV; (ii) female sex; and (iii) BMI < 18.5. Normal‐weight control subjects (n* *= 16) were recruited from School of Pharmacy, University of Oslo, Norway in May 2016. Inclusion criteria for normal‐weight volunteers were (i) female sex; and (ii) BMI ≥ 18.5.

All subjects gave written, informed consent. The study was approved by the Regional Committee for Medical and Health Research Ethics South‐East.

### 2.2 4βOHC analyses

Plasma concentration of 4βOHC was determined by a previously described UPLC‐MS/MS assay.^11^, ^12^ The lower limit of quantification was 10 ng/mL. Intra‐ and interday imprecision and inaccuracy for the method were <15% at 10 ng/mL and <4% at 644 ng/mL (n* *= 6).^11^ All samples were stored at −80°C between sampling and analysis. For the underweight patient samples, stability of 4βOHC during storage was ensured by controlling that 4αOHC concentration was lower than 4βOHC concentration.^13^ This was not done for normal weight samples, since they were analyzed only 5 months after sampling; within the time frame that 4βOHC is known to maintain stability.^14^

### 2.3 Endpoints and statistical analyses

BMI was calculated as body weight divided by height squared (kg/m^2^). 4βOHC/C ratio was calculated as 4βOHC concentration (nmol/L) divided by total cholesterol concentration (mmol × 10^6^/L). Mann‐Whitney *U* tests were used for comparisons of 4βOHC/C ratio and other characteristics between anorexia nervosa patients and normal‐weight subjects. Spearman correlation was used to evaluate association between BMI and 4βOHC/C ratio and between 4βOHC and cholesterol. Statistical significance was considered as *P *< 0.05. GraphPad Prism for Windows, version 6.01 (GraphPad Software, La Jolla, CA), was used for statistical analyses and graphical presentations.

## 3 RESULTS

Clinical and demographic characteristics of included subjects are listed in Table 1. All included subjects were female, and there were no differences in age or total cholesterol between the two groups (*P *> 0.1). None of the included subjects used CYP3A inducers according to the Flockhart CYP Drug Interaction Table.^15^ One of the anorexia nervosa patients used fluoxetine, a CYP3A inhibitor.^15^ The patient was not excluded since results showed higher 4βOHC concentration in underweight patients compared to normal‐weight subjects. All drugs used by included subjects are listed in Table 2.

**Table 1:** Clinical and demographic characteristics

Variables	Anorexia nervosa (n* *= 20)	Normal‐weight (n* *= 16)	*P* value
Age, years	24 (15‐47)	23 (19‐48)	0.81
Body weight, kg	43 (29‐53)	61 (43‐77)	<0.0001
Body mass index, kg/m^2^	14.9 (10.1‐18.0)	21.5 (19.4‐25.2)	<0.0001
Total cholesterol, mmol/L	5.07 (2.83‐6.88)	4.54 (3.44‐5.80)	0.12

Data are expressed as median (range), and *P* values are derived from Mann‐Whitney *U* tests.

**Table 2:** Overview of drugs used by included subjects

Anorexia nervosa patients		Normal‐weight subjects	
Drugs	Number of patients	Drugs	Number of subjects
Alimemazine	1	Cetirizine	2
Chlorprothixene	1	Combination contraceptives	5
Desloratadine	1	Desloratadine	2
Fluoxetine	1	Levothyroxine	2
Levothyroxine	1	Naproxen	1
Melatonin	1	Paracetamol	1
Metoprolol	1	Progesterone only contraceptives	6
Promethazine	1	Valerian root	1
Quetiapine	1		

**Figure 1:**
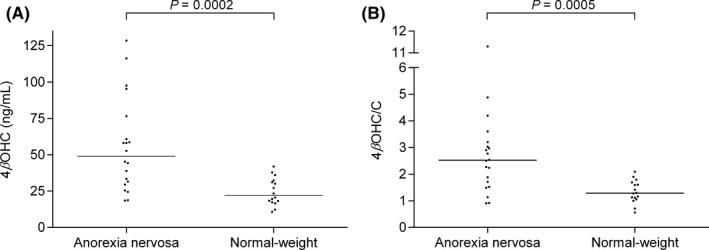
(A) 4β‐hydroxycholesterol (4βOHC) concentration and (B) 4β‐hydroxycholesterol/cholesterol (4βOHC/C) ratio in anorexia nervosa patients (n* *= 20) and normal‐weight subjects (n* *= 16). 4βOHC/C is expressed as molar ratio × 10^−5^. *P* values are derived from Mann‐Whitney *U* tests, and medians are expressed as solid lines

**Figure 2:**
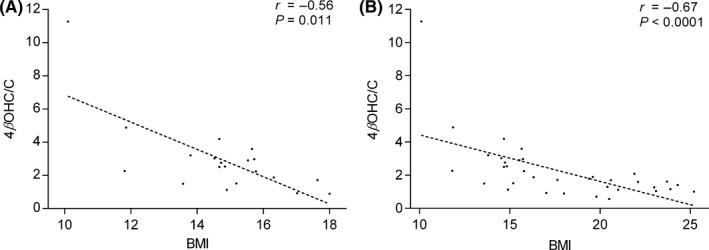
Correlations between 4β‐hydroxycholesterol/cholesterol (4βOHC/C) ratio and body mass index (BMI) in (A) anorexia nervosa patients (n* *= 20) and in (B) the whole study population (n* *= 36). 4βOHC/C is expressed as molar ratio × 10^−5^. *P* and *r* values are derived from Spearman correlations, and linear trend lines are added for visual purposes

The association between 4βOHC and cholesterol concentration in the whole study population was significant (*r *= 0.41, *P *= 0.013). The median 4βOHC concentration in underweight patients was 49.0 ng/mL (range, 18.5–129 ng/mL) compared to 22.0 ng/mL (10.8–41.9 ng/mL) in normal‐weight subjects (*P *< 0.0001) (Figure 1A). Underweight patients had a median 4βOHC/C ratio (molar ratio × 10^−5^) of 2.52 (0.90–11.3) compared to 1.29 (0.56–2.09) in normal‐weight subjects (*P *= .0005) (Figure 1B). 4βOHC/C ratio was negatively correlated with BMI in underweight patients (*r *= −0.56, *P *= .011) (Figure 2A), and in the whole study population (*r *= −0.67, *P *< 0.0001) (Figure 2B).

## 4 DISCUSSION

We found that the underweight patients had twice the plasma 4βOHC/C ratio of normal‐weight volunteers. Furthermore, 4βOHC/C ratio was strongly correlated with BMI in the whole population regardless of the participants’ status as underweight or normal‐weight. This suggests that the negative correlation between 4βOHC/C and BMI, which has previously been reported between 4βOHC/C and body weight in healthy volunteers, extends to underweight patients. The findings indicate that CYP3A activity increases with decreasing BMI, resulting in higher CYP3A activity in underweight patients compared to normal‐weight subjects. However, this needs to be studied further with other CYP3A substrates in underweight vs. normal‐weight individuals.

Studies evaluating CYP3A metabolism in anorexia nervosa patients are scarce. Boyar et al. reported reduced clearance of cortisol, a partial CYP3A substrate, in anorexia nervosa patients compared to healthy subjects.^16^ Cachectic patients have been reported to have both increased and decreased CYP3A metabolism,^17^, ^18^ but not to have altered liver content of CYP3A proteins.^19^ Altogether, conflicting reports make it difficult to conclude whether CYP3A metabolism in underweight patients diverges from normal‐weight subjects.

In this study, we report that anorexia nervosa patients have higher 4βOHC concentration and 4βOHC/C ratio compared to normal‐weight volunteers, and hypothesize that this reflects elevated CYP3A activity in underweight patients. Anorexia nervosa patients often have hypercholesterolemia.^20^ Hyperadiponectinemia is known to occur in anorexic patients,^21^ and may be related to increased cholesterol synthesis.^22^ To account for impact of altered cholesterol levels on 4βOHC concentration, we consider 4βOHC/C ratio to be a more appropriate CYP3A biomarker in this study population. However, whether using 4βOHC concentration or 4βOHC/C ratio, we report approximately twice the biomarker level in underweight patients compared to normal‐weight volunteers. The normal‐weight volunteers had 4βOHC levels and 4βOHC/C ratio within the normal range.^23^ Obesity has been associated with reduced plasma 4βOHC levels,^8^ and hence emaciation could lead to increased 4βOHC levels. However, distorted 4βOHC levels could be caused by changes both in production and elimination, and 4βOHC is further metabolized via CYP7A1 and CYP27A1.^3^ Reduced 4βOHC and elevated plasma 27OHC levels have been reported in an obese mouse model, which could indicate that obesity is associated with increased CYP27A1 activity and therefore increased elimination of 4βOHC.^24^ However, Ulvestad et al. reported strong negative correlation between BMI and both hepatic and small‐intestinal CYP3A protein expression in obese patients,^10^ supporting our findings that CYP3A activity is correlated with BMI.

Theoretically, reduced tissue distribution of 4βOHC and/or increased lipolysis may result in increased 4βOHC/C ratio in anorexia nervosa patients. Thus, it cannot be ruled out that increased 4βOHC/C ratio might be caused by other factors than increased CYP3A activity in this underweight population. However, due to the strong correlation between BMI and 4βOHC/C ratio in the whole study population, including both underweight and normal‐weight subjects, we find it likely that the increased 4βOHC/C ratio reflects an increase of CYP3A activity.

A limitation of the present study is that only one CYP3A biomarker was tested. Unfortunately, no probe drug such as midazolam was given to the participants at the time of the study. Thus, additional studies with other CYP3A substrates are necessary to confirm our results. Genotyping of CYP3A4 and CYP3A5 would also have been of interest. Expression of the CYP3A5*1 allele has been associated with elevated 4βOHC levels,^5^, ^25^ although a larger study did not find any association between 4βOHC levels and CYP3A5*3 or CYP3A4*22 polymorphisms.^2^ Other potential sources of inter‐individual variation in 4βOHC levels are ethnicity and inflammation.^5^, ^26^

An advantage with 4βOHC as biomarker is its selectivity; CYP3A4 and CYP3A5 convert cholesterol to 4βOHC, while only negligible amounts of 4βOHC were produced by seven other CYP enzymes.^3^, ^4^ Furthermore, 4βOHC has an unusually long half‐life of up to 17 days, which leads to low intra‐individual variability.^27^ Since 4βOHC is sensitive to CYP3A induction,^27^ we consider it a suitable biomarker for the present study where anorexic patients displayed elevated 4βOHC levels.

In conclusion, we report that anorexia nervosa patients have twice the plasma 4βOHC/C ratio of normal‐weight volunteers, and that 4βOHC/C ratio has a strong negative correlation with BMI. The findings indicate that CYP3A activity increases with decreasing BMI, resulting in higher CYP3A activity in underweight patients compared to normal‐weight subjects. However, the potential clinical relevance needs to be studied further by comparing pharmacokinetics of drugs subjected to CYP3A‐mediated metabolism in underweight vs. normal‐weight individuals.

## AUTHOR CONTRIBUTIONS

Participated in research design: Hole, Heiberg, Molden. Contributed to acquisition of data: Hole, Heiberg, Gjestad, Mehus, Rø, Molden. Performed data analysis: Hole. Wrote or contributed to the writing of the manuscript: Hole, Heiberg, Gjestad, Mehus, Rø, Molden.

## Disclosure

None declared.
